# Blood volume and hemodynamics during treatment of major hemorrhage with Ringer solution, 5% albumin, and 20% albumin: a single-center randomized controlled trial

**DOI:** 10.1186/s13054-024-04821-6

**Published:** 2024-02-05

**Authors:** François Jardot, Robert G. Hahn, Dominique Engel, Christian M. Beilstein, Patrick Y. Wuethrich

**Affiliations:** 1 Department of Anaesthesiology and Pain Medicine, Inselspital, Bern University Hospital, University of Bern, 3010 Bern, Switzerland; 2 Karolinska Institutet at Danderyds Hospital (KIDS), Stockholm, Sweden

**Keywords:** Plasma volume expansion, Hemorrhage, 5% albumin, 20% albumin, Ringer-lactate, Fluid therapy

## Abstract

**Background:**

Volume replacement with crystalloid fluid is the conventional treatment of hemorrhage. We challenged whether a standardized amount of 5% or 20% albumin could be a viable option to maintain the blood volume during surgery associated with major hemorrhage. Therefore, the aim of this study was to quantify and compare the plasma volume expansion properties of 5% albumin, 20% albumin, and Ringer-lactate, when infused during major surgery.

**Methods:**

In this single-center randomized controlled trial, fluid replacement therapy to combat hypovolemia during the hemorrhagic phase of cystectomy was randomly allocated in 42 patients to receive either 5% albumin (12 mL/kg) or 20% albumin (3 mL/kg) over 30 min at the beginning of the hemorrhagic phase, both completed by a Ringer-lactate replacing blood loss in a 1:1 ratio, or Ringer-lactate alone to replace blood loss in a 3:1 ratio. Measurements of blood hemoglobin over 5 h were used to estimate the effectiveness of each fluid to expand the blood volume using the following regression equation: blood loss plus blood volume expansion = factor + volume of infused albumin + volume of infused Ringer-lactate.

**Results:**

The median hemorrhage was 848 mL [IQR: 615–1145]. The regression equation showed that the Ringer-lactate solution expanded the plasma volume by 0.18 times the infused volume while the corresponding power of 5% and 20% albumin was 0.74 and 2.09, respectively. The Ringer-lactate only fluid program resulted in slight hypovolemia (mean, − 313 mL). The 5% and 20% albumin programs were more effective in filling the vascular system; this was evidenced by blood volume changes of only + 63 mL and − 44 mL, respectively, by long-lasting plasma volume expansion with median half time of 5.5 h and 4.8 h, respectively, and by an increase in the central venous pressure.

**Conclusion:**

The power to expand the plasma volume was 4 and almost 12 times greater for 5% albumin and 20% albumin than for Ringer-lactate, and the effect was sustained over 5 h. The clinical efficacy of albumin during major hemorrhage was quite similar to previous studies with no hemorrhage.

*Trial registration*: ClinicalTrials.gov NCT05391607, date of registration May 26, 2022.

## Background

Treatment of major hemorrhage is a challenge for the clinician whenever it occurs in the prehospital setting, in the operating theatre, and in the intensive care unit (ICU). Although blood transfusion is preferred in uncontrolled hemorrhage (trauma patients), hemorrhage in better controlled environments is treated with “clear fluids” until a pre-specified blood hemoglobin level (“transfusion trigger”) is reached. Intravenous fluid administration during surgery should still be planned to maintain normovolemia and adequate hemodynamics. However, fluid management is more challenging than in the awake state, as general anesthesia obtunds the body´s ability to correct both fluid overload and hypovolemia; the diuretic response to infused fluid is only 10–15% of normal and the depressive effect of intravenous and volatile anesthetics on lymphatic pumping promotes hypovolemia and decreases the ability to compensate hypovolemia by capillary refill [1–5].

Therefore, well-performed fluid therapy requires knowledge about both the hemodynamic adaptation to general anesthesia and to the blood volume (BV) expanding properties of available fluids in scenarios where surgery is associated with major hemorrhage.

Animal models provided evidence in understanding the mechanistic effect of fluid administration in hypovolemic shock [6, 7]. However, quite few randomized studies compare the clinical efficacy of crystalloid and colloids fluid during major hemorrhage in humans, and they have usually been focused on trauma cases [8]. Because crystalloids quickly equilibrate between the intravascular and interstitial volume, very large fluid volumes are needed if only crystalloid fluids are used, which increases the risk of overfilling the patients, raising complications like pulmonary edema, re-bleeding due to dilution effect, and poor healing [9]. Smaller volumes are believed to be needed when colloid fluids are used, which is due to their different pharmacokinetic profile. Dextran and hydroxyethyl starch have left the market, leaving albumin as the colloid fluid of choice in the ICU and in the perioperative setting, but its documentation is mainly based on old literature [10]. The hyperoncotic 20% albumin is known to offer strong plasma volume expansion (PVE) per infused volume, being approximately twice the infused volume, in volunteers [11] and during surgery with minor hemorrhage [12]. However, the efficacy of albumin solutions during major surgery has been questioned based on the belief that the capillary leakage rate is markedly increased by shedding of the endothelial glycocalyx layer [13].

The plasma volume expansion (PVE) effects of albumin solutions have not been adequately studied during surgery associated with major hemorrhage. The primary aim of this randomized clinical trial study was to compare the PVE properties of iso-oncotic 5% albumin, hyper-oncotic 20% albumin, and RL as the fluid therapy during major intraoperative hemorrhage situations. Open radical cystectomy was chosen as study model, which is associated with blood loss of 500–1200 mL during the 1/2–1 h when the bladder is removed in the middle part of the operation [14–19].

The primary hypothesis was that 20% albumin has the strongest PVE effect in this setting. We also hypothesized that adding a pre-defined amount of 5% or 20% albumin to crystalloids would better maintain the BV compared to using a crystalloid alone program in the 3:1 ratio to the blood loss.

## Methods

### Ethics approval

The study was approved by the Swiss government’s local ethics committee (cantonal ethics committee KEK Bern, Switzerland, KEKBE ID 2022–00209, chairperson Prof C. Seiler, May 11, 2022), prospectively registered at ClinicalTrials.gov (NCT05391607, principal investigator P.Y. Wuethrich, May 26, 2022), and conducted in accordance with the good clinical practice recommendation of the Declaration of Helsinki. Preoperative written informed consent to participate was obtained according to local regulations. Reporting complied with the recommendations of the Consolidated Standards of Reporting Trials (CONSORT) statements. The full trial protocol can be accessed on request.

### Study design, inclusion and exclusion criteria

This was an investigator-initiated, open-label, three-arm, randomized, active controlled single-center trial conducted at the Department of Urology of the University Hospital Bern, Switzerland, between May 26, 2022, and April 13, 2023.

Consecutive patients planned for pelvic lymph node dissection, open radical cystectomy, and urinary diversion were screened for inclusion. Inclusion criteria were age > 18 years old and non-emergency surgery. Exclusion criteria were renal dysfunction (eGFR < 60 mL min^−1^ 1.73 m^−2^, heart failure, hypersensitivity or allergy to exogenous albumin, pregnancy, breastfeeding, known or suspected drug or alcohol abuse.

### Anesthesia and preparations

All patients were encouraged to drink 800 mL of a carbo-loading solution (Preload, Nestlé Health Science, Vevey, Switzerland) the evening before and 400 mL up to 2 h before arrival at the operating theater. No i.v. fluids were administered within 12 h before surgery.

A thoracic epidural catheter was placed before induction of anesthesia and epidural analgesia maintained during the surgery with bupivacaine 0.25% at an infusion rate of 6 to 8 mL/h. Anesthesia was induced with fentanyl 2 µg/kg, propofol 2 mg/kg, and rocuronium 0.6–0.9 mg/kg intravenously to facilitate endotracheal intubation and maintained with sevoflurane 0.6 MAC (age corrected) and dexmedetomidine at a rate of 0.3 µg kg^−1^ h^−1^. Ventilation with an inspired oxygen fraction of 60% was mechanically controlled to maintain P_arterial_CO_2_ between 35 and 40 mmHg, with a positive end-expiratory pressure of 5 mmHg and tidal volume of 8 mL/kg according to our standards. Supportive administration of norepinephrine to maintain a target MAP of > 65 mmHg was initiated at a rate of 0.03 µg kg^−1^ min^−1^.

### Fluid programs

Patients were allocated by randomization to one of three volume replacement groups: Ringer-lactate (RL) (group “Ringer”), 5% albumin (group “5% albumin”), 20% albumin (group “20% albumin”).

**Fig. 1:**
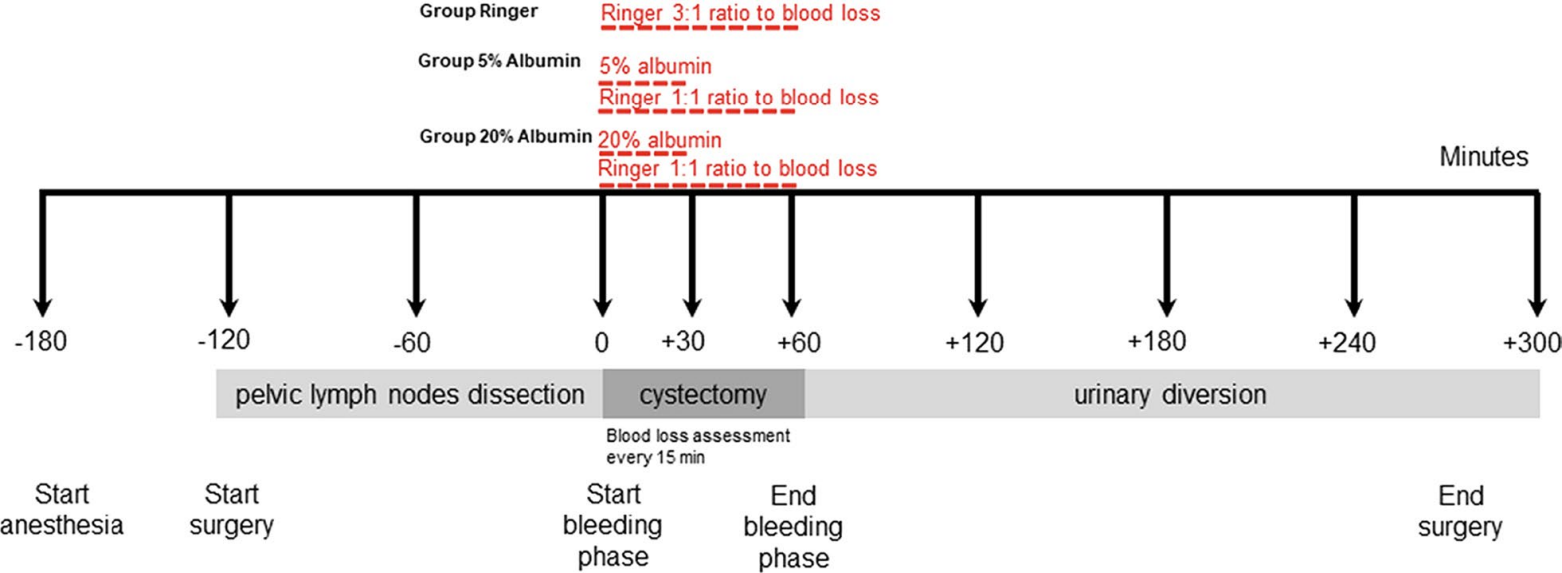
Timeline of the procedure

1. RL (Fresenius Kabi AG, Kriens, Switzerland) was administered in the active control group “Ringer.” RL solution was administered at a 3:1 ratio of the blood loss [6, 20]. The timeline for the experiments is illustrated in Fig. 1.
2. 5% albumin (Albumin CSL 5%, CSL Behring, Bern, Switzerland) was given at a volume of 12 mL/kg (ideal body weight), was started, and continued over 30 min at a constant rate during removal of the bladder [21].
3. 20% albumin (Albumin CSL 20%, CSL Behring, Bern, Switzerland) 3 mL/kg (ideal body weight) was started and continued over 30 min at a constant rate during removal of the bladder [11]. Both albumin infusions were complemented by a RL matching blood loss in a 1:1 ratio [21].

A baseline intravenous fluid administration was performed using 1 mL kg^−1^ h^−1^ of RL solution during the pelvic lymph node dissection (1st part of surgery) and during the removal of the bladder (2nd part of surgery, known as the hemorrhagic phase). The baseline substitution rate was then increased to 3 mL kg^−1^ h^−1^ during the last part (3rd phase) until the end of surgery [22, 23].

Fresh frozen plasma transfusion was administrated in the presence of continuous excessive microvascular bleeding (relative indication based on the senior surgeons’ observations), and packed red blood cells were transfused to maintain a blood hemoglobin (Hb) value > 80 g/L.

Blood loss was assessed every 15 min by accounting for the aspirated blood and the weight difference of the gauzes before and after use at regular intervals [24].

### Blood chemistry

Venous blood (5 mL) was drawn from the proximal lumen of the central venous catheter just before (time 0 min) and at 10, 20, 30, 40, 50, 60, 75, 90, 120, 180, 210, 240, and 300 min after the start of the bladder removal phase. Before each sampling, a small volume of blood was drawn from the central venous catheter to avoid dilution effects and re‐administered after the sampling was finished and flushed with 2 mL of 0.9% saline to prevent clotting.

Routine analyses of blood samples were conducted in the hospital's central laboratory. The Hb concentration was sampled in EDTA tubes and analyzed on a Sysmex XN (Sysmex Corp, Kobe, Hyogo, Japan) with a coefficient of variance 0.7%, as ensured by duplicate samples drawn at baseline.

### Hemodynamics

Cardiac output (CO) and the mean arterial pressure (MAP) were recorded with a CardioQ esophageal probe (Deltex Medical, Chichester, UK) at the same time as the blood samples were collected. A radial arterial catheter was inserted to monitor the MAP.

The central venous pressure (CVP) was measured via a cannula placed in the right internal jugular vein shortly after the induction of anesthesia after calibration against the atmospheric pressure prior to the induction of anesthesia.

### Outcomes

We aimed to monitor the PVE as the primary outcome over 300 min and mathematically separate the clinical efficacy of RL solution from those of 5% albumin and 20% albumin solutions when used to combat hypovolemia during removal of the bladder (hemorrhagic phase of cystectomy).

Cardiac output served as secondary outcome, while the other registered secondary outcomes will be reported elsewhere. Exploratory analyses included MAP and CVP values, the half-lives (T_1/2_) of the intravascular PVE for the RL and the albumin solutions, and the fluid balance in the morning of postoperative day (POD) 1.

### Calculations

#### Blood volume

The present study used Hb dilution to estimate the BV changes during the surgery. Radioisotopes have been used to validate this mode of BV monitoring without and with blood loss [25, 26]. The calculations assume an even distribution of Hb in the BV.

Two mathematical approaches were used to evaluate PVE based on Hb dilution [27]. The first one was a regression method which studies the linear relationships between PVE, blood loss, and the cumulative volumes of infused fluid. The measurements from all time points were pooled into a single analysis [28, 29].

The calculation of PVE was performed by allowing the plasma dilution to be corrected for blood sampling and hemorrhage [26, 30, 31]. The latter is known to occur to approximately 3/4 of the total surgical bleeding during the period when the bladder was removed (hemorrhagic phase) [19]. The calculations were performed as follows: The initial BV was first obtained using the isotope-derived formula by Nadler et al. [32], where the total BV prior to the infusions (BV_0_) was derived from the height (*h*) in meters, body weight (*w*) in kilograms, and sex:

$$\begin{gathered} {\text{Male}}:{\text{BV}}_{0} = 0.3669h^{3} + 0.03219w + 0.6041 \hfill \\ {\text{Female}}:{\text{BV}}_{0} = 0.{3561}h^{{3}} + 0.0{33}0{8}w + 0.{1833} \hfill \\ \end{gathered}$$

The BV at a later time 1 was calculated by first estimating the total hemoglobin mass in the circulation at baseline [Hb_mass(0)_] as being equal to the product of BV_0_ and the blood Hb concentration at baseline, Hb_o_. Losses from Hb_mass_ were then subtracted for each measurement, and the BV_1_ obtained by dividing this difference by a freshly taken Hb [26, 33].

$$\begin{aligned} & {\text{Hb}}_{{{\text{mass}}(0)}} = {\text{BV}}_{{\text{o}}}\times {\text{Hb}}_{{\text{o}}} \\ & {\text{Hb}}_{{{\text{mass}}({1})}} = {\text{Hb}}_{{{\text{mass(}}0{)}}} - {\text{Blood}}\;{\text{loss}}_{{\left( {0 - {1}} \right) }} \left[ {\left( {{\text{Hb}}_{0} + {\text{Hb}}_{{1}} } \right)/2} \right] \\ & {\text{BV}}_{{1}} = {\text{Hb}}_{{{\text{mass}}({1})}} /{\text{Hb}}_{{1}} \\ & {\text{Blood}}\;{\text{volume}}\;{\text{expansion}}\;\left( {{\text{BVE}}} \right) = {\text{BV}}_{{1}} - {\text{BV}}_{{\text{o}}} \\ \end{aligned}$$

This procedure was subsequently repeated for each measurement, and the BV was re-estimated. The result was then compared to the amount of each type of infused fluid and the estimated volume of blood lost up to that point in time. The PVE properties of RL and albumin were given by the factors X and Y in the following multiple regression equation:

$${\text{PVE}} + {\text{blood}}\;{\text{loss}} = {\text{factor}} + {\text{X}}\;{\text{volume}}\;{\text{of}}\;{\text{albumin}} + {\text{Y}}\;{\text{volume}}\;{\text{of}}\;{\text{RL}}$$

The second mathematical approach was the area method. The area under the volume–time curve (AUC) was calculated with the linear trapezoid formula for the BV changes between 0 and 300 min for each patient separately. The AUC for the blood loss was calculated in a similar way, assuming that the loss of 1 mL of blood decreased the BV by 1 mL. The expected AUC for each infusion of RL was based on the BV expanding property for RL obtained by the regression analysis. The AUC for 5% and 20% albumin solution was then obtained as the sum of the AUC for the blood loss (positive) and AUC for BVE (negative in hypovolemia) minus AUC for the RL solution (positive).

The plasma volume-expanding power of RL was finally obtained as the AUC divided by the product of the infused volume and the length of the observation period (300 min).

The half-life of the PVE attributable to 5% and 20% albumin was calculated based on the terminal part of the volume-time curves, which were derived mathematically by deducting off the regression-derived PVE for RL.

### Randomization and power analysis

Randomization was performed using a computer-generated list with blocks of 14 patients; all patients were block randomized after inclusion into either intervention group 5% albumin or 20% albumin solution or the active control group Ringer in a 1:1:1 ratio according to random numbers generated by computer allocation. Blinding for the involved anesthesiologists was not considered possible, but patients and data investigators were blinded.

The mean PV expansion at the end of an infusion of 12 mL/kg of 5% albumin solution in volunteers was 576 ± 140 mL, based on data from Zdolsek and Hahn [21]. We hypothesized that 5% albumin would be 30% more effective than RL in proportion 3:1 in compensating blood loss (effect size 576–443/140 = 0.92). With a power of 75% and significance of *P* < 0.05 yielded N = 14 in each group.

### Statistics

The data are presented as the mean ± standard deviation and changes evaluated by the paired *t*‐test or nonparametric repeated-measures analysis of variance (RM ANOVA) if normally distributed. The data are presented as the median (interquartile range, IQR) if skewed, and changes are evaluated by the Mann–Whitney U test. Correlations between parameters were analyzed by simple linear regression. A *P* value of < 0.05 was considered statistically significant for global statistical tests.

Fifteen-minute measurement averages of PPV and MAP were calculated for the average of the start of surgery (08:15 and 13:30) and for the bleeding part of the surgery (5-min intervals between 10:00 and 12:00). An RM ANOVA was conducted for the changes in PPV and MAP over time [34]. In case of significance, *post-hoc* tests were performed using RM ANOVA for paired and the Mann–Whitney test for independent groups.

A few time points with missing data were deleted from the regression analysis and the AUC calculations, but the mean value of the precedent and following measurement was used when RM ANOVA was applied.

*P*-values < 0.05 were considered statistically significant. The analyses were conducted with the statistical software R, version 4.0.2; R Foundation for Statistical Computing, Vienna, Austria. URL https://www.R-project.org/ except where noted.

## Results

**Fig. 2:**
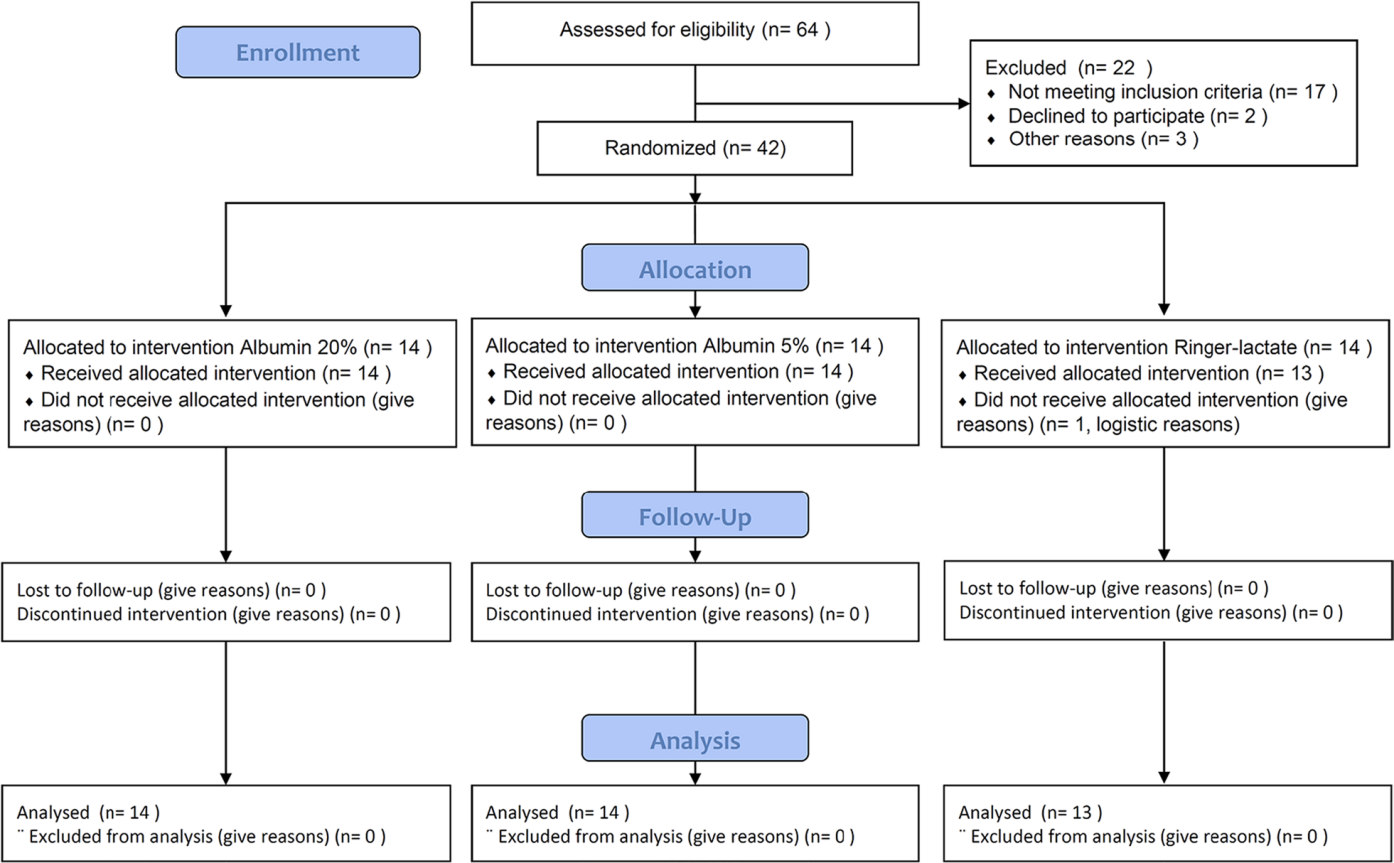
CONSORT flow chart

Of 64 consecutive patients scheduled for cystectomy, 22 were excluded and 42 were randomized (Fig. 2). The pre-operative baseline characteristics were similar between groups (Table 1).

**Table 1:** Baseline characteristics presented as median [IQR] or incidence (%)

Variable	Ringer	5% albumin	20% albumin
Age (y)	68 [57, 84]	62 [57, 68]	66 [62, 70]
BMI (kg/m^2^)	23.5 [22.8, 27.8]	23.4 [21.2, 28.3]	24.4 [21.6, 30.4]
Sex (female/male)	5/8 (39% / 61%)	5/9 (36% / 64%)	2/12 (14% / 86%)
ASA (2/3/4)	10/2/1 (77%/15%/8%)	12/2/0 (86%/14%/0%)	7/7/0 (50%/50%/0%)
Ischemic heart disease	0/13 (0%/100%)	1/13 (7%/93%)	0/12 (0%/100%)
Hypertension	9/4 (69%/31%)	7/7 (50%/50%)	7/7 (50%/50%)
Diabetes mellitus	1/12 (8%/92%)	0/14 (0%/100%)	3/11 (21%/79%
B-Hb (g/L)	124 [116, 130]	130 [112, 142]	129 [116, 143]
Plasma albumin (g/L)	34 [32, 39]	37 [33, 38]	36 [34, 37]
Plasma creatinine (µmol/L)	81 [70, 94]	81 [72, 97]	86 [74, 105]
GFR (mL/min)	78 [69, 89]	83 [65, 90]	82 [65, 89]

*ASA* American Society of Anesthesiologists physical status classification system, *B-Hb* blood hemoglobin value, *GFR* glomerular filtration rate

### Infused volumes

The infused volume of 5% albumin and 20% albumin solution was 884 (± 215) mL (mean ± SD) and 239 (± 48) mL, respectively. The median administered RL solution was 2.8 L in the Ringer group, 1.8 L in the 5% albumin, and 2.0 L in the 20% albumin, *P* = 0.003 (pairwise post hoc test: 5% albumin vs RL: *P* = 0.002 and 20% albumin vs RL: *P* = 0.005). Overall, the blood loss (including amount of blood lost due to blood samplings) did not differ significantly between the groups (*P* = 0.90); (Table 2). Three patients received blood components. These volumes were counted as iso-oncotic fluid and placed in the same column as 5% albumin. Two patients in the 5% albumin group had a shorter operating time than 5 h (3.5 and 4 h, respectively).

**Table 2:** Fluid balance variables during surgery and in the first postoperative morning

	Ringer	5% albumin	20% albumin	P-value
*Surgery*				
Duration of surgery (min)	325 [302, 377]	347 [336, 404]	343 [321, 373]	0.487
Hemorrhagic phase (min)	64 [51, 80]	56 [45, 69]	58 [50, 75]	0.836
Blood loss (mL)	694 [601, 1306]	862 [600, 1095]	832 [749, 1165]	0.900
Crystalloids (mL)	2800 [2350, 4200]	2182 [1500, 2400]*	2000 [1700, 2300]*	0.003
Albumin (mL)	0	884 ± 215	239 ± 48*	< 0.001
PRBC (yes)	2/11 (15%/85%)	0/14 (0%/100%)	0/14 (0%/100%)	0.339
FFP (yes)	1/13 (8%/92%)	0/14 (0%/100%)	1/13 (7%/93%)	0.338
Norepinephrine (mg)	1.52 [0.74, 2.60]	1.12 [0.79, 1.17]	1.01 [0.75, 1.25]	0.218
*Postoperative Day 1*				
Total fluid IN (first 24 h)	5250 [4430, 6550]	5100 [4580, 5570]	4695 [4330, 5200]	0.417
Total fluid OUT (first 24 h)	2850 [1980, 3230]	2730 [2145, 3270]	2245 [1830, 2740]	0.182
Fluid balance (first 24 h)	2610 [1330, 3995]	2335 [1630, 2540]	2525 [2025, 2940]	0.731
Change in body weight (kg)	1.90 [1.00, 3.10]	0.75 [-0.10, 1.90]	0.90 [0.50, 1.70]	0.100
B-Hb (g/L)	85 [79, 99]	85 [83, 98]	84 [72, 92]	0.709

Data are given as the median [IQR], mean ± SD, or incidence (%)

*P < 0.002 compared to Ringer group; ^+^ *P* < 0.002 compared to 5% albumin

*PRBC* packed red blood cells, *FFP* fresh frozen plasma, *B-Hb* blood hemoglobin value, *GFR* glomerular filtration rate

### Plasma volume expansion

**Fig. 3:**
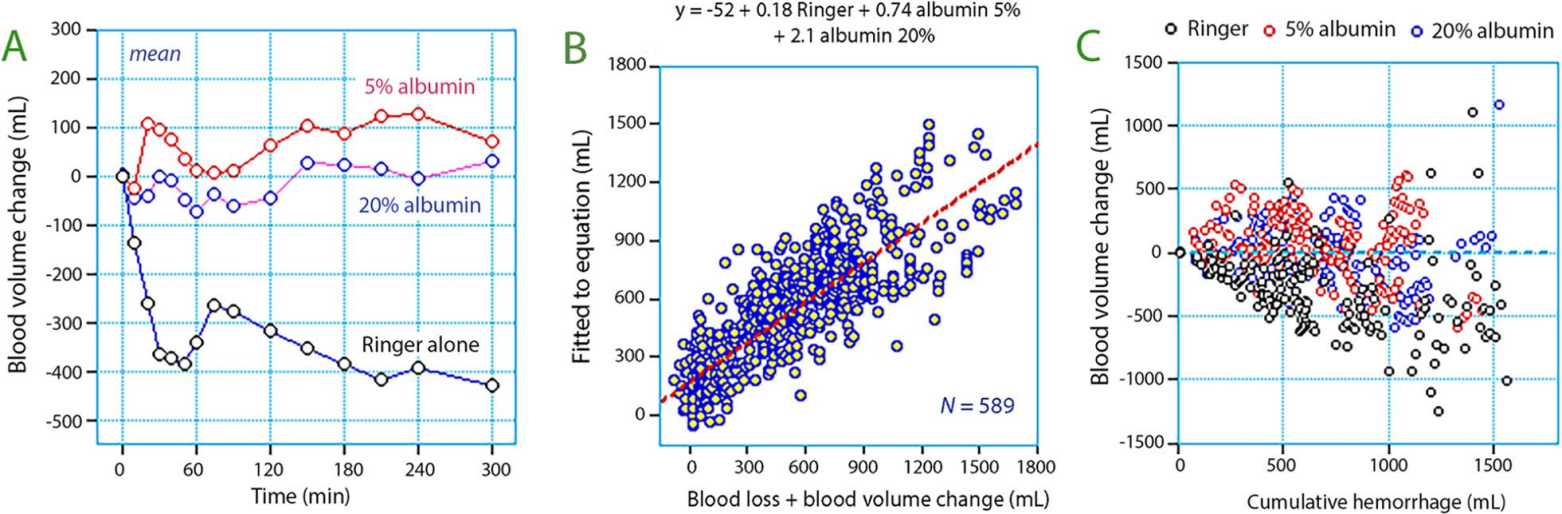
**A** Blood volume trends for the three infusion strategies. Mean values are shown. **B** Multiple regression analysis used to obtain the plasma volume expanding power of the three infusion fluids. The equation is shown on top. **C** Blood volumes changes according to the cumulating hemorrhage illustrates the overall pattern of hypovolemia/hypervolemia in the study

**Fig. 4:**
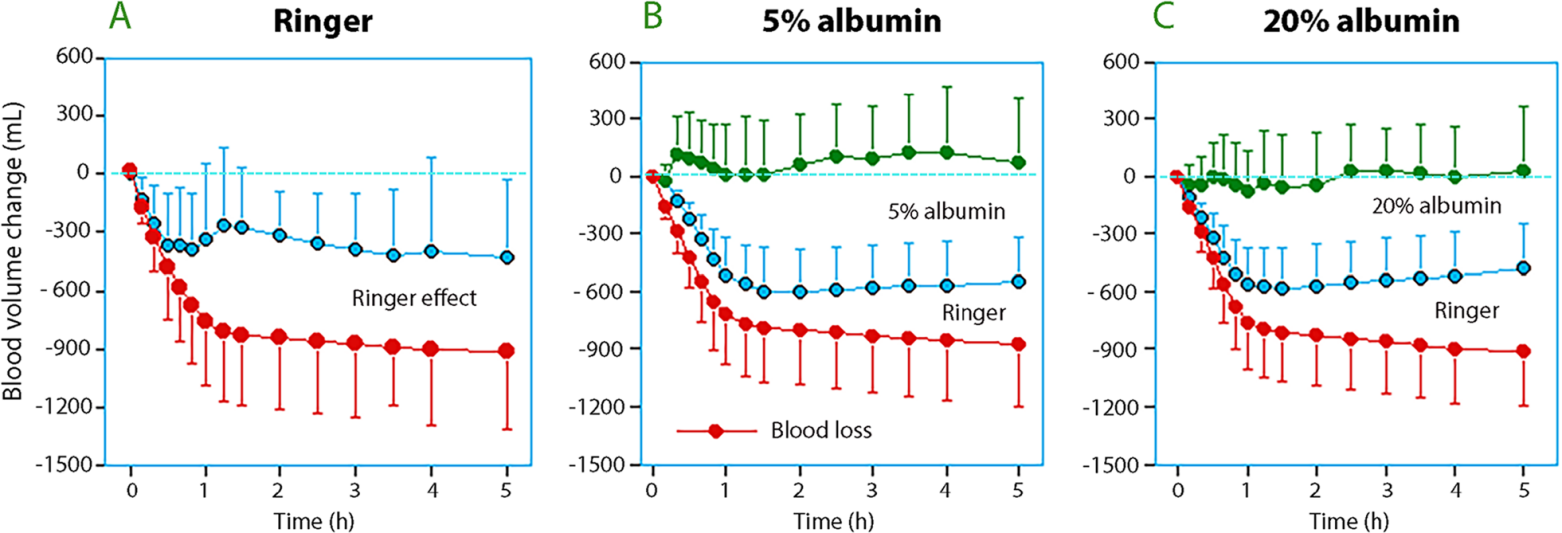
Blood loss and the volume contribution of each infusion fluid to how well the BV was maintained. The top curve in each subplot represents the BV change. The data are the mean with standard deviation

The BV in the three treatment groups is shown in Fig. 3A and 4. The mean BV change was − 313 (± 226) mL in the Ringer group, + 63 (± 227) mL in the 5% albumin group, and − 44 (± 229 mL) in the 20% albumin group (*P* < 0.001; Scheffe post hoc test showed that the Ringer group had lower BV than the albumin groups). These differences remained statistically significant at the end of the study (*P* < 0.0002) and also when the analyses were remade without the three patients who received blood components (*P* < 0.001). RM ANOVA based on all BV estimates confirmed the existence of a statistically significant difference between the groups without time or interaction effects (*P* < 0.001). A negative BV change of > 500 mL at the end of surgery occurred in 31% of the patients in the RL-alone group but in none of the patients who received albumin (Fig. 3C).

### Plasma volume expanding properties

The regression method showed strong correlation (r = 0.825) between the blood loss (positive) plus the BVE (negative in hypovolemia) and the independent effects of the infused volumes of RL solution and 5% and 20% albumin solution (*P* < 0.0001).

The mean PVE effect was 0.18 (95% CI, 0.17–0.21) for RL, 0.74 (0.67–0.79) for the 5% albumin solution, 2.09 (1.87–2.28) for the 20% albumin solution (*P* < 0.001). The complete analysis is shown in Table 3 and the regression analysis further illustrated in Fig. 3B. The median residual error of the regression was − 11 mL and the median absolute residual error 132 mL.

**Table 3:** Independent PV expanding effects when Ringer-lactate was administered together with 5% and 20% albumin during surgery

Count 588	r^2^ = 0.681			*P* < 0.0001	
	Beta coefficient table				
	Best estimates	95% CI	Standard error	t-value	*P*-value
Intercept	− 51.747				
Ringer	0.178	0.175–0.213	0.009	20.409	< 0.0001
5% albumin	0.735	0.674–0.785	0.772	29.690	< 0.0001
20% albumin	2.094	1.873–2.280	0.590	22.963	< 0.0001

The best estimates were equal to the factors obtained in a multiple regression analysis where the infused volumes of the three fluids at each time point were related to the sum of the blood loss and the calculated BV expansion (regression method)

Based on AUC, RL had a PVE power of only 0.06–0.08 times the infused volume when the entire surgical period was considered. The PVE power of 5% albumin solution remained at 0.76 while 20% albumin solution expanded the PV by 2.10 times the infused volume.

### Cardiac output

**Fig. 5:**
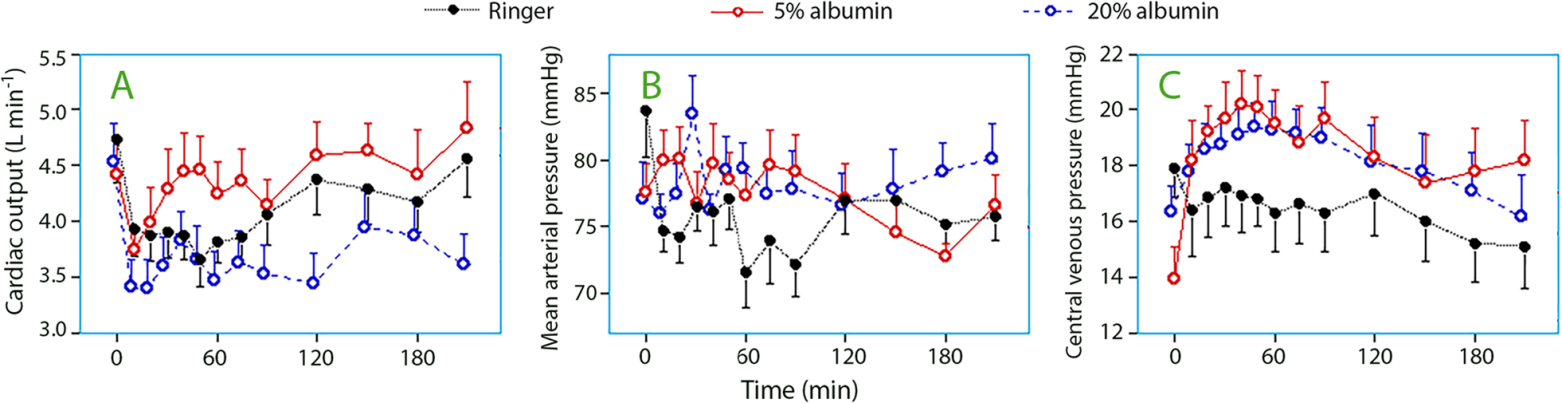
Cardiac output (**A**), mean arterial pressure (**B**), and central venous pressure (**C**). The data are presented with the standard error of the mean

CO briefly decreased in the early phase of the hemorrhage, increased, however, after correction over the entire study period by + 0.1 (mean, ± SD 0.7) L/min in the Ringer group, + 0.7 (± SD 0.7) in the 5% albumin group, and + 0.3 (± SD 0.8) L/min in the 20% albumin group (based on all data points, *P* < 0.001, Scheffé post hoc test shows by *P* < 0.05 that Ringer < 5% albumin, and 5% albumin > 20% albumin) (Fig. 5A).

### Explorative analyses

MAP did not change significantly during the interventions, but the Ringer group had a transient decrease between 60 and 90 min (*P* < 0.01, ANOVA and Scheffé test, Fig. 5B). The CVP increased during the administration of the albumin solutions (both *P* < 0.001), but not when RL was administered (P = 0.33; Fig. 5C).

**Fig. 6:**
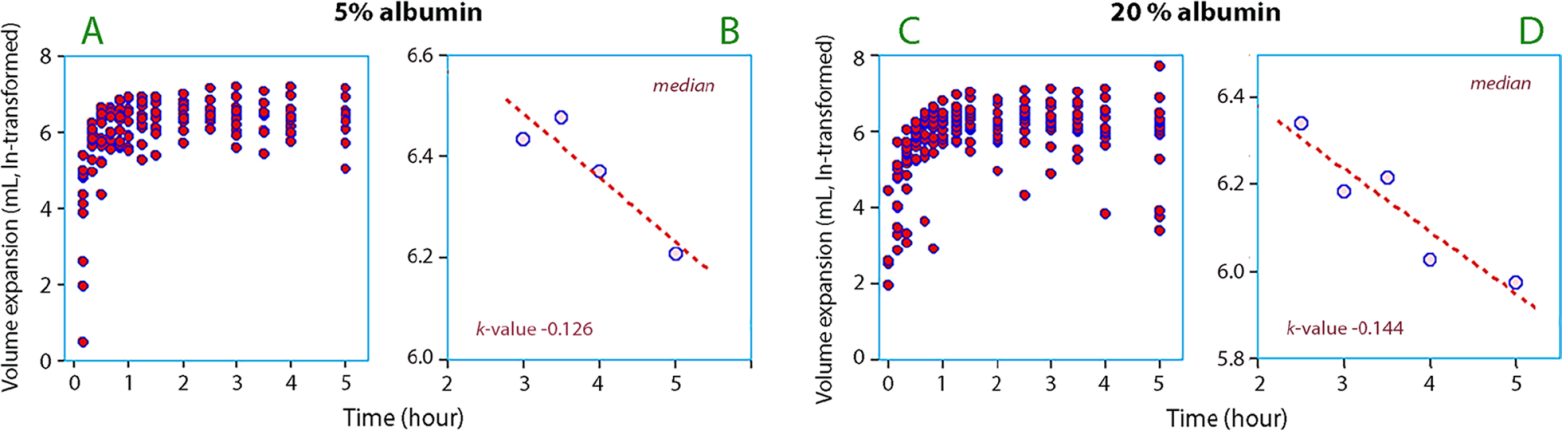
Crude data used the calculation of T_1/2_ for 5% and 20% albumin are shown in subplots (**A**) and (**C**). The T_1/2_ were then obtained as the k-value for the regression slope connecting the mean values for the ln-transformed the volume expansion over time. **B** The *k*-value for 5% albumin was − 0.126, which corresponds to a T_1/2_ of 5.5 h. **D** The *k*-value for 20% albumin was − 0.144, which corresponds to a T_1/2_ of 4.8 h

The median T_1/2_ for the PVE was 5.5 h of 5% albumin and 4.8 h for 20% albumin solution (Fig. 6). The terminal T_1/2_ for the PVE of RL averaged 3.9 h, but the true baseline was then prolonged by the baseline administration of RL.

Under-substitution with fluid was more likely when bleeding was large. Exploratory analysis was performed based on 74 data points (from a total of 790) associated with rapid bleeding (total blood loss already > 500 mL and Hb loss > 1 g/min). Here, the average BV was 200 mL below baseline and unevenly distributed between the groups: RL − 465 (± 388) mL, 5% albumin − 86 (SD ± 225) mL and 20% albumin − 119 (± 183) mL (*P* < 0.001, Scheffeest Ringer vs the albumin groups, *P* < 0.001).

The increase in body weight from the preoperative value to the first postoperative morning was 1.91 (± 0.33) kg in the Ringer group, 1.08 (± 0.35) kg in the 5% albumin group, and 0.85 (± 0.36) kg in the 20% albumin group (*P* = 0.100).

## Discussion

### Key results

The present study used surgical blood loss as model to compare fluid treatments in the case of major hemorrhage. RL was infused to combat hypovolemia in a 3:1 proportion to the blood loss while a fixed dose of 0.6 g/kg of albumin was given either as 5% or 20% albumin solution combined with RL in a 1:1 proportion to the blood loss.

The results show that the PVE power of the fluids differed greatly: The PVE power was 0.18 for RL, 0.73 for 5% albumin, and 2.09 for 20% albumin, confirming our primary hypothesis that 20% albumin has the strongest PVE effect as a fluid therapy during hemorrhage. Only the PVE power of RL decreased markedly during the 300-min study period, illustrating the long intravascular persistence of the albumin solutions. However, the reported PVE power for the fluids is surprisingly similar to those previously reported for crystalloid fluid and for the albumin solutions in volunteers, on POD1 after major surgery, and during surgery with minor hemorrhage. Jacob et al*.* reported a PVE power of 0.17 for RL in volunteers [35], and studies of albumin have yielded a PVE power of 0.66 for 5% albumin and 2.00 [21] and 1.80 [36] for 20% albumin. Surgery associated with minor hemorrhage yielded a PVE power of 1.7 for 20% albumin [12] while it was 1.9–2.2 on POD1 after major surgery [36]. Ernest et al*.* found the PVE power to be 0.52 for 5% albumin when infused after cardiac surgery [37]. Statkevicius et al*.* reported a PVE power of 0.65–0.74 at 3 h after initiating infusions of 5% albumin at different rates during major abdominal surgery, which illustrates the long-lasting PVE induced by albumin solutions [38]. Lamke and Liljedahl found that that 50 g of albumin expanded the PV by 500 mL regardless of being administrated as a 5%, 20%, or 25% solution [10].

The hemodynamic profile and the BV calculations showed that the RL-alone treatment was associated with modest hypovolemia (mean, − 313 mL). This finding was further supported by the observation of a transient hypotension at 60–90 min after the onset of the bleeding phase. The albumin solutions better filled the vascular system but also increased the CVP, which suggests that they were often “non-responders” to fluid loading [39].

The clinical efficacy of the 5% albumin solution was slightly better than the 20% albumin solution due to the greater BV expansion and higher CO. This finding could be explained on the fact that the 5% albumin infusions consisted of much larger fluid volume than 20% albumin did (884 *versus* 239 mL).

Finally, body weight change at POD 1 was approximately 1 kg greater in the RL group compared to both albumin groups.

### Blood volume changes

The results support the value of using 5% or 20% albumin concomitant with the administration of RL to maintain a long-lasting intraoperative normovolemia [24, 40, 41]. The 5% albumin solution showed slightly better results in terms of PVE as compared to 20% albumin, which could be due to the larger infused volume. Interestingly, the results do not support that the PVE properties of albumin solutions are attenuated during major surgery. The PVE attributable to the albumin solutions showed a median T_1/2_ of approximately 5 h, which is somewhat shorter than the 6.5 h to 7.6 h for 5% and 20% albumin recently found in volunteers [21, 36]. However, this does not necessarily imply that transcapillary leakage of albumin was accelerated, as a short T_1/2_ could have been promoted by leakage of fluid and albumin into the surgical field.

When considering the entire surgical period, the PVE was negative (− 313 mL) in patients treated with RL alone and thereby received a less efficient fluid therapy compared to both albumin groups. This trend was most apparent during ongoing major bleeding (see Fig. 3c). The regression equation seems to exaggerate the need for fluid when the hemorrhage is small while somewhat underestimating the need for fluid when the hemorrhage is large. This was further studied by dividing the interventions into 1-h periods; the PVE properties were approximately 25% weaker during the first hour as compared to the entire study period. One explanation could be losses of fluid into operating field. From the second hour, the BV expanding properties of the both albumin solutions had gained their final strength while RL showed gradually lower values. Here, the reason could be that RL became distributed and excreted faster than the albumin solutions.

One of the purposes of infusing albumin instead of crystalloid fluid is to reduce the positive postoperative fluid balance. In the present study, the perioperative gain in body weight up to POD1 was almost twice as large in the RL alone group than in the albumin groups. This supports our view that combined administration of RL and albumin is a valuable option in terms of fluid management in major surgery involving intestinal anastomosis and in patients who are at risk of delayed return of gastrointestinal function. [9, 42]. However, the numerical difference in postoperative weight gain did not quite gain statistical significance.

CO increased slightly in the albumin groups while the BV remained unchanged, which might be due to dissociation between these parameters due to hypovolemic stress [43]. These differences should be regraded with judicious caution as the patients in the Ringer group received more norepinephrine to maintain the target MAPs. However, the difference was not statistically significant.

### Treatment of major hemorrhage

The ultimate treatment of hemorrhage is to transfuse whole blood or blood components. Reasons why blood is withheld during surgery until the indication is very strong is that clear fluids are associated with fewer adverse effects. Moreover, a restrictive blood transfusion practice has been related to better outcomes after cystectomy in patients with urothelial carcinoma [44]. If the bled volume is not compensated, the adult human responds by increasing systemic vascular resistance, which is mainly due to secretion of norepinephrine. This reaction maintains MAP while CO decreases as there is less blood to pump [20, 43, 45]. Recruitment of interstitial fluid to the plasma by “capillary refill” is initiated but operates too slowly to compensate for major hemorrhage [25, 46]. When the bleeding exceeds 1 L, the body changes strategy from vasoconstriction to vasodilatation, whereby MAP suddenly drops. This reason for this changeover is unknown but might occur at an earlier stage during anesthesia; experiments in sheep show that capillary refill is seriously blunted by volatile anesthetics, which is probably due to an inhibitory effect on lymphatic pumping [2, 3]. In our studies, the early administration of norepinephrine could possibly have counteracted the blunted physiological reaction to hemorrhage from our anesthetics.

Volume treatment by infusion fluid that matches the blood loss to restore all physiological responses to hypovolemia provided that irreversible shock has not yet developed. Crystalloids such as RL or saline solution are the standard treatment for more-than-minimal hypovolemia. Support for this practice from animal studies [6, 7, 19], volunteer experiments, and trauma studies [47]. In addition, a large body of literature has accumulated over the past decades that evaluates the usefulness of hypertonic or hyperoncotic solutions to combat hypovolemic states, but these approaches are not currently favored [45, 48, 49]. The nature of the optimal fluid solution, amount, and timing of administration are still matter of debate [20, 43, 45]. Target endpoints for fluid resuscitation are also still debated and undefined. A too aggressive fluid replacement could result in hypervolemia and rebleeding [6, 7, 19].

The rationale behind the administration of albumin solutions is an expansion of the blood volume and a maintenance of normovolemia with reduced positive fluid balance due to significant reduction in crystalloid fluids. Albumin solutions also possess a long-lasting effect over at least 5 h. Albumin administration impairs the blood coagulation but less than for other colloids [50] and, compared to RL, does not increase the hemorrhage during cystectomy [51].

The concomitant administration of albumin solution in a 5% or 20% concentration to a restricted administration of RL suggests a formulation with a potent efficacy of conventional resuscitation therapy in hemorrhage and continuous bleeding for a period of around 1 h, without the administration of additional fluids, reducing positive fluid balance. This is of importance as excessive postoperative fluid balance has been related to poorer outcome including postoperative complications like anastomotic leakage in major surgery involving intestines or colon [52, 53]. In addition, our study shows that a replacement in a 3:1 ratio with RL alone resulted in a modestly reduced blood volume.

Finally, as guidelines for the transfusion of packed red blood cells (PRBC) generally follow restrictive thresholds (60–70 g/L for asymptomatic healthy patients, 80 g/L in patients with coronary artery disease or according to the guidelines and clinical trials on transfusion requirements in critical care 70 g/L as the threshold for critically ill patients, adequate and longstanding PVE are required and considered as the first line treatment before administering PRBC [54, 55]. This is of importance as the intraoperative administration of PRBC has been related to worse cancer related outcome in cystectomy patients [44].

### Limitations

The clinical efficacy of infusion fluids can be assessed in several ways, none of which is perfect in all situations. Isotope dilution requires a steady state with regard to the fluid balance during the equilibration period, which can be difficult to guarantee [56]. The present approach is forgiving for errors in time and yet offers robust answers in complex scenarios with ongoing hemorrhage and simultaneous infusion of two fluids having different characteristics.

The starting point for the calculations was the BV at the time when the hemorrhagic phase began. Anesthesia induction and the first part of the surgery might have promoted slight hemodilution, but this was counteracted by the infusion of norepinephrine. However, the erythrocyte pool, which was the biomarker used for the calculations, was largely unaffected by the events occurring before the studied infusions were initiated.

The amount of infused fluid in the RL alone group was titrated to match the bled volume in the proportion 3:1. By contrast, albumin was administered as bolus infusions over 30 min when a known phase of hemorrhage started. The strategies to administer fluid study arms were not fully congruent, but we believe that our protocol well reflects how the two types of fluid would be provided in the clinical situation. Moreover, there is no widely accepted rule for how much 20% albumin should be infused to combat major blood loss during surgery. However, we could find a well-adapted therapy as we could maintain normovolemia with the infused amount.

Our “bleeding” model is focused on a controlled hemorrhage model. The clinical context of uncontrolled hemorrhage, like traumatic bleeding, demands a different approach that includes early transfusion of blood, optimization of coagulation, and acceptance of a sub-normal MAP [6].

The present study is the first to separate the PVE properties of different infusion fluids when administered simultaneously during ongoing intraoperative hemorrhage. Our approach might contribute to the implementation of effective intraoperative fluid management concepts in the future, but whether this will result in better clinical outcomes remains speculative and a matter for further studies.

## Conclusions

Plasma volume expansion averaged 0.2 times the infused volume of Ringer-lactate, 0.7 times the infused volume of 5% albumin, and 2.1 times the infused volume of 20% albumin during major surgery with hemorrhage of approximately 800 mL. Both 5% and 20% albumin were potent plasma volume expanders and the effect was longstanding. By contrast, the replacement ratio of 3:1 ratio was not sufficient to maintain normovolemia in the Ringer-lactate alone group, suggesting only limited plasma volume expansion with a replacement ratio of 3:1.

## Data Availability

The datasets used and/or analyzed during the current study are available from the corresponding author on reasonable request.

## Abbreviations

GFR: Glomerular filtration rate
BVE: Blood volume expansion
PVE: Plasma volume expansion
MAP: Mean arterial pressure
CO: Cardiac output
POD: Postoperative day
i.v.: Intravenous
BV: Blood volume
Hb: Hemoglobin
AUC: Area under the curve
SD: Standard deviation
BMI: Body mass index
FFP: Fresh frozen plasma
PRBC: Packed red blood cells
SVR: Systemic vascular resistance
ROC: Receiver operating characteristics
AUC: Area under the curve
PRBC: Packed red blood cells
RL: Ringer-lactate
